# Variability in Observation-based Onroad Emission Constraints from a Near-road Environment

**DOI:** 10.3390/atmos11111243

**Published:** 2020-11-18

**Authors:** Heather Simon, Barron H. Henderson, R. Chris Owen, Kristen M. Foley, Michelle G. Snyder, Sue Kimbrough

**Affiliations:** 1 Office of Air Quality Planning and Standards, US EPA, RTP, City, 27711, NC; 2 Center for Environmental Measurement and Modeling, US EPA, RTP, 27711, NC; 3 Wood Environment and Infrastructure Solutions, Inc., Durham, City, 27703, NC

**Keywords:** near-road measurements, mobile source emissions, CO, NO_x_, linear regression, top-down constraints

## Abstract

This study uses Las Vegas near-road measurements of carbon monoxide (CO) and nitrogen oxides (NO_x_) to test the consistency of onroad emission constraint methodologies. We derive commonly used CO to NO_x_ ratios (ΔCO:ΔNO_x_) from cross-road gradients and from linear regression using ordinary least squares (OLS) regression and orthogonal regression. The CO to NO_x_ ratios are used to infer NO_x_ emission adjustments for a priori emissions estimates from EPA’s MOtor Vehicle Emissions Simulator (MOVES) model assuming unbiased CO. The assumption of unbiased CO emissions may not be appropriate in many circumstances but was implemented in this analysis to illustrate the range of NOx scaling factors that can be inferred based on choice of methods and monitor distance alone. For the nearest road estimates (25m), the cross-road gradient and ordinary least squares (OLS) agree with each other and are not statistically different from the MOVES-based emission estimate while ΔCO:ΔNO_x_ from orthogonal regression is significantly higher than the emitted ratio from MOVES. Using further downwind measurements (i.e., 115m and 300m) increases OLS and orthogonal regression estimates of ΔCO:ΔNO_x_ but not cross-road gradient ΔCO:ΔNO_x_. The inferred NO_x_ emissions depend on the observation-based method, as well as the distance of the measurements from the roadway and can suggest either that MOVES NO_x_ emissions are unbiased or that they should be adjusted downward by between 10% and 47%. The sensitivity of observation-based ΔCO:ΔNO_x_ estimates to the selected monitor location and to the calculation method characterize the inherent uncertainty of these methods that cannot be derived from traditional standard-error based uncertainty metrics.

## 1. Introduction

Understanding the magnitude of air pollution emissions across a variety of sources is critical to air quality management. The characterization of emissions from all sources is referred to as an emissions inventory. In the United States, the Environmental Protection Agency (EPA) publishes a National Emissions Inventory (NEI) once every three years, which includes annual emissions of criteria pollutants and toxic pollutants by county [[1]]. Emissions inventories can be constructed using either bottom-up or top-down methodologies.

Bottom-up inventories are based on detailed information about emissions factors (mass of emitted pollutant per unit of activity, e.g., grams of carbon per gallon of fuel burned), and their dependence on source characteristics (e.g., vehicle speed, vehicle age, etc.), and activity data (e.g., fuel burned, vehicle miles traveled, vehicle idling time, etc.) [[2–6]]. Emissions from individual sources are then aggregated to create an inventory. For mobile source inventories in the United States, models such as EPA’s MOtor Vehicle Emissions Simulator (MOVES) [[7]] and California’s Emission FACtors (EMFAC) [[8]] model are used to simulate the range of driving conditions and vehicle classes on different road types and can be employed at fine scales such as road segments, at larger scales such as county-level aggregates or using nationwide defaults. MOVES parametrizations are based on laboratory and field study empirical relationships, which have been scaled up to the nation, to capture mean vehicle performance for a variety of vehicles classes, ages, and driving conditions. The accuracy of MOVES emissions estimates depends upon the inputs provided and the fitness of the empirical relationship for the location (e.g., by state, by city, by intersection).

Top-down inventories use measurements of ambient composition to quantify the amount of mass emitted into the atmosphere [[3,9–13]]. These inventories generally provide information about emissions from groups of sources (e.g., mobile sources) rather than detailed information on individual sources. Top-down approaches are often used to adjust bottom-up inventories, but top-down adjustments using different types of measurements may not always agree with each other [[14–16]].

Top-down constraints often rely on relative concentrations to implicitly account for decreased concentrations farther from a source due to dispersion and transport. One common method is to look at the ratio of two pollutants as a signature for certain emissions sources. For example, the ratio of carbon monoxide (CO) to the nitrogen oxides (NO_x_ = NO + NO_2_) has been used to identify dominant source contributions [[17–22]] and to evaluate bottom-up emissions estimates [[23–32]]. In order to isolate a source or group of sources, these studies generally measure the enhancement of each pollutant over reference concentrations, often denoted as ΔCO:ΔNO_x_. Specifically, an upwind measurement may be used to estimate the background CO and NOx concentrations (i.e., the reference concentration) for an emissions source, while the difference between the background and downwind measurements can provide the incremental increase of each pollutant due to the emission source [[33]]. Alternatively, linear regressions have been used to infer ΔCO:ΔNO_x_ from measurements made at a single location [[23–32]] which is then interpreted to represent emissions from a nearby source.

Previous studies have used ambient data to estimate ΔCO:ΔNO_x_ resulting from mobile sources that can then be used to infer the emitted CO:NO_x_ ratio from this source category. To isolate a signal from onroad mobile sources some studies use near-road/tunnel measurements [[27,30,32]] or measurements made during morning rush-hour [[19,24,25,28,29,31,34]], while others have relied on ambient daytime measurements [[17,18,21,23]]. Simon et al. [[35]] explored the limitations of interpreting ΔCO:ΔNO_x_ values not derived from near-source environments for the purpose of constraining emissions from a specific source category. Those results suggest that photochemical aging, such as photochemical CO production, and deposition could complicate the quantification of emission factors based solely on ambient measurements when measurements are not made in close proximity to the source.

Here we further explore the value of regression-based methods for constraining mobile source emissions estimates using a comprehensive near-road measurement study in Las Vegas [[36]]. We applied two methods for inferring CO:NO_x_ emission ratio: the regression method using data from a single near-road monitor and a dual-monitor, upwind-downwind subtraction technique. Since many near-road analyses rely on data from a single monitor to collect data representing air masses impacted by the roadway [[27,30,32]] (i.e., neither upwind-downwind relationships nor measurements at multiple distances from the roadway are available), we endeavor to evaluate the uncertainty in observation-based ΔCO:ΔNO_x_ ratios by comparing values derived from multiple methods using monitors at multiple downwind distances. The inferred emission ratios are then used to characterize the potential uncertainty in constraining bottom-up mobile source emissions estimates derived using MOVES.

## 2. Methods

In the methods section we begin by describing the Las Vegas near-road study site location and measurements (Section 2.1). In section 2.2., we describe data screening criteria applied, which were intended to select for conditions where the downwind monitors predominantly captured impacts from the I-15 traffic and experience minimal impacts from other nearby sources. We then describe the various ambient data analysis methods used to derive ΔCO:ΔNO_x_ from these near-road measurements, including the cross-road gradient (Section 2.3.1) and two regression methodologies (Section 2.3.2). Finally, we describe MOVES simulations that were used to compute bottom-up vehicle CO and NO_x_ emissions estimates based on traffic information from I-15 (Section 2.4) for comparison against the top-down estimates derived using methods from Section 2.3.

### 2.1. Las Vegas Near-Road Field Study

The measurements and design of the Las Vegas near-road field study have been described previously [[36,37]] and key details are summarized below.

The measurement sites were located near a section of Interstate 15 on the South side of Las Vegas, which runs exactly north-south. There were 4 monitors aligned approximately perpendicular to the roadway including one monitor that was 135m upwind (west) of the interstate and 3 monitors downwind (east) of the interstate at varying distances: 25m, 115m, and 300 m (see figure S1). The measurement sites were also adjacent to a golf course and the 300 m downwind site was approximately 435 m from the boundary of the McCarran International Airport. Data were collected continuously between December 2008 and February 2010. Concentration data for CO, NO, and NO_2_ were logged at 5-min intervals at each site. A summary of instrument specifications and uncertainties have been summarized elsewhere [[36]] and Table S1 provides manufacturer specifications. All CO and NO_x_ monitors were subject to nightly span and zero checks using calibration gas. The NO_x_ zero checks were stable, typically varying by 0.1 ppb over 24 h and a single systematic shift of 1.5 ppb between October and December 2010, well within the requirements for regulatory monitors (3.1 ppb). The span checks were also stable indicating a typical drift of 0.5% over 24 h, and a range of ±5% over the full time period, also within regulatory monitoring requirements (10.1%). The CO checks showed similar compliance, with an average zero drift of 10 ppb over 24 h and of 50 ppb over the year (versus the requirement of 410 ppb) and a typical span drift of 0.2% over 24 h and of 2.5% over the full duration of the study (versus the requirement of 10.1%). Onsite meteorology data were also collected at the field site.

### 2.2. Data Screening Criteria

We begin by screening our one-hour data to only include conditions in which the monitors are truly in an upwind/downwind configuration such that the downwind monitors should predominantly represent air coming from the interstate. First, we filtered our data to only include hours which had 1-hr average westerly wind direction (between 230° and 300°) and which had a wind speed greater than 1 m/s as has been done previously with this dataset [[36]]. We further restricted the data by eliminating any hours for which the wind was not in a westerly direction for one or more 5-min average periods. A wind rose for the period of December 2008 through December 2009 is provided in Figure S2. Finally, we confirmed that there were no remaining hourly datapoints for which the upwind concentration of CO or NO_x_ was larger than the concentrations at the 25m downwind site.

Several additional screening criteria were applied to check for other conditions of concern. First, we checked whether any hourly CO or NO_x_ concentrations at the 300m downwind site were larger than the corresponding hourly concentration at the 25m downwind site. This would indicate that sources other than the interstate might be contributing to the 300m downwind site. A small subset of data was removed based on this check. Additionally, we removed any hourly data that were below the instrument minimum detection limit (25 ppb for CO and 0.5 ppb for NO_x_).

Zero and span points quality assurance checks, which were often performed in the middle of the night, usually resulted in only 4 valid 5-min averages within a one-hour period. To eliminate measurements made during instrument calibration and testing and to ensure that we included enough datapoints for robust regression estimation, we did not analyze any hours at a particular monitor that had fewer than 6 valid 5-min average measurements (50% completeness) of either CO or NO_x_.

Over the 15-month study, there were over 7500 h with valid CO and NO_x_ measurement data at all monitors. Less than 20% of the measurements had wind direction in a westerly direction with 1 m/s wind speeds and of those less than 20% had at least 6 5-min averages with valid measurements. Overall, 145 h met all screening criteria set out above. Sample size by season and time of day is shown in Table S2.

For each of these 145 hours, we calculated the cross-road gradient ΔCO:ΔNO_x_ using hourly average CO and NO_x_ mixing ratios (Section 2.3.1). Within each hour, we used 5-min average concentrations at downwind monitors to supply the datapoints used in the linear regressions (Section 2.3.2). For each hour of data, we performed a separate analysis for each of the three downwind monitors such that there are 3 cross-road gradient calculations (one for each downwind monitor) and 6 regression slopes (2 regression methods for each of 3 downwind monitors).

### 2.3. Ambient Data Analysis Methods

We take advantage of the multi-monitor set-up for this field study to calculate ΔCO:ΔNO_x_ using several different methods. The first method (Section 2.3.1) utilizes measurements made at the upwind and downwind monitors to calculate the cross-road gradient to quantify incremental change in CO and NO_x_ resulting from roadway sources. For the second method (Section 2.3.2), we use linear regression of data from individual near-road monitors to estimate ΔCO:ΔNO_x_ using two different regression techniques. These two types of methods provide different estimates of ΔCO:ΔNO_x_ based upon spatial gradient using multiple instruments (cross-road gradient, Section 2.3.1) and temporal variability measured by a single instrument (regression slope, Section 2.3.2). The observationally-derived ΔCO:ΔNO_x_ values can then be used to constrain bottom-up MOVES-based NO_x_ emissions estimates from nearby I-15 roadway sources assuming that CO emissions are unbiased.

#### 2.3.1. Upwind Monitor Data to Determine Cross-Road Gradient ΔCO:ΔNO_x_

Equation 1 is used to calculate ΔCO:ΔNO_x_ from the cross-road gradient, where the DW subscript represents concentrations measured at one of the downwind monitors and the UW subscript represents concentrations measured at the upwind monitor:

(1) $$\frac{E_{CO}}{E_{{NO}_{x}}}\approx \frac{\Delta CO}{\Delta {NO}_{x}}=\frac{{CO}_{DW}-{CO}_{UW}}{{NOx}_{DW}-{NOx}_{UW}}$$

This method relies on the assumption that the major difference between measured concentration at the upwind and downwind monitors come from emissions along the I-15 roadway and therefore $\frac{\Delta CO}{\Delta {NO}_{x}}$ can be interpreted as an estimate of the emitted ratio of CO to NOx ($\frac{E_{CO}}{E_{{NO}_{x}}}$). While data selection for wind-speed and direction (see Section 2.2) are intended to optimize for this condition, several other factors may impact differences in measured concentrations including differences in calibration of the upwind and downwind monitors, impacts from other nearby sources, and local-scale wind patterns (see Section 5 for further discussion). The largest distance between upwind and downwind monitors is 400m. With a minimum wind-speed requirement of 1 m/s (see Section 2.2), air parcels will take less than 7 min to travel from the upwind to the downwind monitor. Deposition and chemical loss lifetimes for both NO_x_ and CO are substantially longer than 7 min, (hour to days) for NO_x_ [[38–43]] and weeks to months for CO [[44–47]] and are not expected to be important in measured differences between monitors.

#### 2.3.2. Regression Analyses for Determining Roadway ΔCO:ΔNO_x_

Here we use the linear relationship between observed CO and NO_x_ mixing ratios at a downwind monitor to calculate ΔCO:ΔNO_x_. *CO*_*UW*_ and *NOx*_*UW*_ can be replaced by constant background concentrations (*CO*_*BG*_ and *NOx*_*BG*_) and Equation 1 can be rearranged as follows:

(2) $${CO}_{DW}=\frac{\Delta CO}{\Delta {NO}_{x}}\times {NOx}_{DW}+({CO}_{BG}-\frac{\Delta CO}{\Delta {NO}_{x}}\times {NOx}_{BG})$$

If *CO*_*DW*_ is plotted versus *NOx*_*DW*_ at co-located monitors, $\frac{\Delta CO}{\Delta {NO}_{x}}$ represents the slope and $\left({CO}_{BG}-\frac{\Delta CO}{\Delta {NO}_{x}}\times {NOx}_{BG}\right)$ represents the intercept. Note that both *CO*_*BG*_ and *NOx*_*BG*_ must be constant over the 1-hour sampling time period for the intercept to be a constant value.

**Figure 1.**
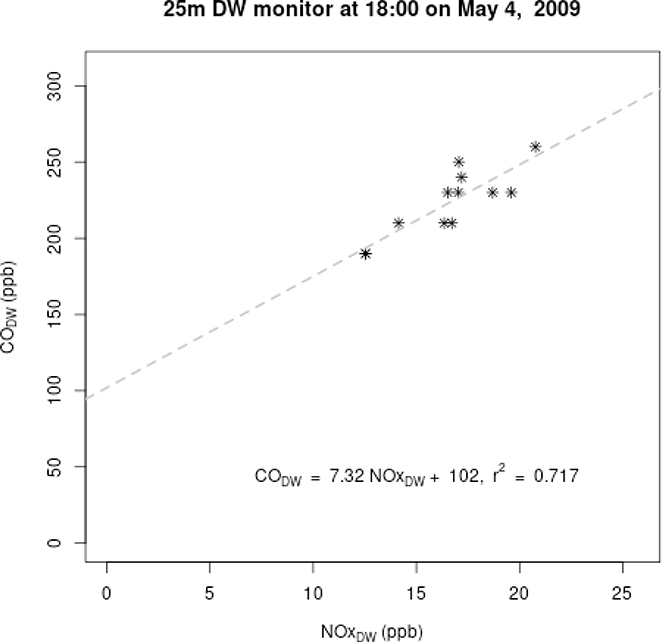
Example of CO:NO_x_ ordinary least squares (OLS) regression methodology using 5-min-averaged measured concentrations to derive change (slope) in emissions for a one-hour increment of time.

To demonstrate, Figure 1 shows the strong linear relationship between 5-min average CO and NO_x_ measurements at the 25m downwind monitor over a 1-hr period. The black dashed line is the estimated regression line, where *CO*_*DW*_ is the response variable and *NOx*_*DW*_ is the explanatory variable. The slope of the regression line (7.32) is interpreted as the incremental increase over background levels in CO divided by the incremental increase in NO_x_ and the intercept (102) is ${CO}_{BG}-\frac{\Delta CO}{\Delta {NO}_{x}}\times {NOx}_{BG}$ Figure S3 in the supplemental information shows that the linear relationship for 5-minute average CO and NO_x_ concentrations persists when looking across the entire dataset used in this study.

There are several linear regression estimation methods that may be appropriate for this type of analysis, depending on the characteristics of the data. Ordinary least squares (OLS) estimation minimizes the average vertical distance between datapoints and the line of best fit and have been used in previous studies to estimate ΔCO:ΔNO_x_ [[23,28,34]]. OLS regression is appropriate either when there is no uncertainty in the explanatory variable (NO_x_ mixing ratios) or when the uncertainty in the response variable (CO mixing ratios) is substantially larger than the uncertainty in the explanatory variable. If these assumptions are not met, the OLS slope estimates can be biased toward zero [[48]]. Another limitation of OLS is that it is not invariant to exchange of the explanatory (x) and response (y) variables (i.e., the slope derived by swapping the x- and y-variables would not be the inverse of the original slope) [[49]]. Thus, the emissions adjustment derived from regressing CO on NO_x_ would be different from regressing NO_x_ on CO. An alternate estimation methodology that has been used in previous studies to determine ΔCO:ΔNO_x_ is called orthogonal regression [[23,29]]. Orthogonal regression minimizes the average perpendicular distance between datapoints and the regression line rather than the vertical distance and is invariant to exchange of x and y. Orthogonal regression is appropriate when the error variance in the response variable is the same as, or very close to, the error variance in the explanatory variable. When this assumption is not met, the orthogonal regression slopes can be biased high [[48]]. Table 1 provides a summary of the error in measurement assumptions for the cross-road gradient and the two regression approaches. For the measurements used in this study, the manufacturer reported NO_x_ instrument error is 0.5 ppb equating to 1–8% for the range of measured concentrations and the CO instrument error is 0.5% equating to 0.7–2ppb for the range of measured concentrations in this study. Actual measurement error is likely to be larger in real-world operating conditions. As noted in section 2.1, span drift for NO_x_ and CO were on the order of 2.5% and 5%, respectively, across the entire measurement period. In this analysis we compare OLS and orthogonal regression to the cross-road approach to explore the impact of these different assumptions and approaches on the estimation of CO to NO_x_ ratios. All analyses described here are carried out using the R statistical language [[50]]. The OLS regressions were performed using the R *stats* package and the orthogonal and regressions were performed using the R *deming* package using the default options. Both of these algorithms estimate standard errors on the slope estimates. These standard errors provide a means of accounting for uncertainty in the regression estimation. We utilize this uncertainty information by only reporting regression-based ΔCO:ΔNO_x_ estimates for statistically significant slopes (*p* < 0.1) which ranged from 29–55% of the regressions depending on method and monitoring distance (Table S3).

### 2.4. Roadway CO:NO_x_ from MOVES Emissions Model Simulations

In this section we describe MOVES 2014a [[51]] simulations and inputs that were used to create bottom-up emissions estimates of CO and NO_x_ for the 145 hours analyzed using the near-road ambient data. These MOVES based estimates of emitted CO:NO_x_ ratios are then available for comparison against estimates based on ambient measurements described in section 2.3.

MOVES highway running emissions rates were calculated using the distribution of vehicle types and ages obtained from a University of California Riverside license plate study in Las Vegas in 2010 [[52]] and hourly ambient temperatures as measured at the airport. Vehicle traffic data for different classes of onroad vehicles are important inputs for the MOVES simulations. For traffic inputs we used 15-min speed and traffic count data and hourly-average vehicle type (heavy-duty vs light-duty) information derived from this field study as described in the paragraph below. The fleet mix used for the MOVES simulations varied by hour, with the distribution between heavy-duty and light-duty vehicles based on traffic measurements made at the site and the distribution of vehicle types within the light-duty and heavy-duty categories based on the UC Riverside license plate study conducted in the area shortly after the field study. This approach is detailed further elsewhere [52,53]. MOVES mass-based emissions were converted to molar ratios of CO to NO_x_ using molecular weights of 28 g/mol for CO and 46 g/mol for NO_x_ since MOVES reports NO_x_ as NO_2_ equivalents.

Traffic data at the measurement site were collected by the Nevada Department of Transportation using Wavetronix 10.525 GHz frequency modulated continuous wave radar units [[36]]. The system was designed to measure vehicle counts in each lane at a 15-min interval, with vehicle counts separated into 10-m length bins. Vehicles less than 30 ft long were characterized as light-duty and longer vehicles were characterized as heavy-duty. While the field study did not record gasoline versus diesel vehicle traffic, light-duty and heavy-duty characterizations were used as a surrogate. Analyses of the traffic data suggest there are missing traffic measurements, measurements with suspiciously low volumes, and measurements with suspiciously low speeds for the measurements made on the northbound lanes. To develop a more realistic emissions profile for the northbound that incorporates known traffic data from the good traffic data, we developed an emissions profile based on data from the southbound lanes and two nearby permanent Nevada Department of Transportation traffic measurement sites located north and south of the measurement site along I-15. The emissions profile is based on the annual average daily traffic (AADT), with the AADT for the northbound lanes determined by multiplying the AADT from the southbound lanes to the ratio of the southbound traffic to the northbound traffic observed at two permanent traffic counters located approximately 5.3 miles to the north and 3.3 miles to the south, resulting in an AADT of 91,264 for the northbound lanes, based on an AADT of 93,535 from the southbound lanes. The AADT for the northbound lanes was used with the average weekday and weekend speed and traffic count based on monthly averages of the traffic from the southbound lanes to determine an hourly traffic count that varied by hour of day, day of week, and month of year. Additionally, we created profiles for the heavy-duty percentage based on vehicle length-based measurements taken simultaneously with the traffic volume measurements in the southbound lanes. Finally, vehicle speeds were also found to have a diurnal pattern on weekdays and weekends; this speed pattern was used for hourly speeds when determining emission rates.

## 3. Results

**Figure 2.**
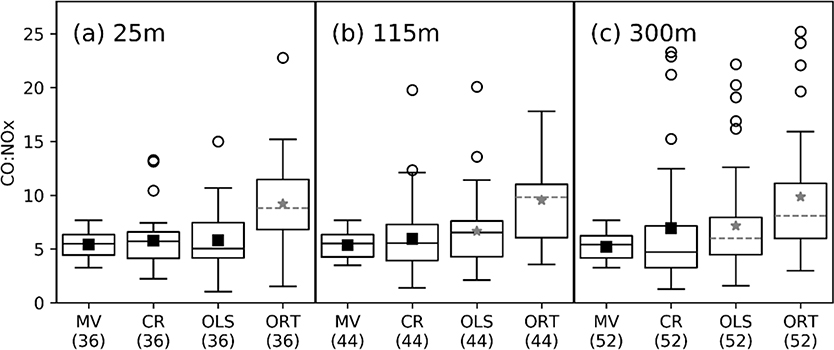
Comparison of emitted CO:NO_x_ from EPA’s MOtor Vehicle Emissions Simulator (MOVES) with ΔCO:ΔNO_x_ values from 3 ambient-based methods. Distribution of emitted CO:NO_x_ from MOVES (“MV”), from the cross-road gradient method (“CR”), from OLS regressions and from orthogonal regressions (“ORT”) at the 25m downwind monitor (**a**), the 115m downwind monitor (**b**) and the 300m downwind monitor (**c**). Numbers below each boxplot represent sample size (n). Boxes represent interquartile range; mid-lines represent median values; and symbols represent mean values. When the Mann Whitney test is statistically different from MOVES, the median line is gray and dashed. When the Welch’s t-test is statistically different from MOVES, the mean is a star and gray. Note that there are 3 outlier points off the scale in the plot: ORT at 115m with a ΔCO:ΔNO_x_ of 30.5; CR at 300m with a ΔCO:ΔNO_x_ of 44.4; ORT at 300m with a ΔCO:ΔNO_x_ of 37.3.

ΔCO:ΔNO_x_ derived from each downwind monitor distance and using each method (nine estimates) were applied to constrain the aggregated MOVES hourly NO_x_ emissions estimates assuming that CO was unbiased. Variability in inferred top-down NO_x_ emissions constraints was used to characterize uncertainty in these ambient-based methods for adjusting NO_x_ emissions. Figure 2 compares the distributions of emitted CO:NO_x_ from MOVES and the nine ambient estimates of ΔCO:ΔNO_x_ across the 145 h meeting our screening criteria. Figure S4 displays these same data as a scatterplot allowing the comparison of MOVES ratios with observationally-derived ratios paired in time. Mean and median values for all methods and monitors are provided in Table S4. Populations of ambient-based ratios are all compared to MOVES ratios using the Mann Whitney U test and the Welch’s t-test. Both tests were performed using *p* < 0.05 as the statically significant threshold. The Welch’s t-test was used because the observationally derived ratios and modeled emission variance are not expected to be similar, since the variance of emissions ratios will not be altered by physical/chemical processing. Since regression results were only included in the dataset when slopes were statistically significant (*p* < 0.1), the regression-based ΔCO:ΔNO_x_ datasets contain fewer than 145 h. For each downwind monitor, only the hours for which we were able to calculate valid ΔCO:ΔNO_x_ values using all three methods are included in the figure. Since the set of hours with valid slopes for both regression methods are slightly different at the three downwind monitors, the subset of hours included in Figure 2 differs between the three downwind distances.

Observationally-derived ΔCO:ΔNO_x_ is lowest from the cross-road gradient and highest from the orthogonal regression. Using data from the 25m monitor, ΔCO:ΔNO_x_ from the cross-road gradient and from OLS regression are not statistically different from MOVES or from each other. Measurements made further downwind (115m and 300m) generally increase the ΔCO:ΔNO_x_ estimates from OLS but not from the cross-road gradient. Consequently the ΔCO:ΔNO_x_ from the cross-road gradient is not statistically different from MOVES estimates of emitted CO:NO_x_ at any of the downwind monitors. OLS regression, on the other hand shows mixed results at 115m downwind (the Welch’s t-test is statistically different with a p-value of 0.02 but the Mann Whitney U test is not with a *p*-value of 0.06) but is statistically higher than MOVES using measurements from 300m downwind. ΔCO:ΔNO_x_ from the orthogonal regressions from all downwind monitor locations is significantly higher than emitted CO:NO_x_ from MOVES and also higher than estimates from the cross-road gradient method or from OLS regressions. As a function of monitor location, all methods vary by 3–30%. The skew of the distribution and number of outliers increases with distance from the road for all methods, which likely influences the MOVES comparison using the mean.

Table 2 provides scaling ratios for MOVES NO_x_ emissions estimates that would be derived based on each method/monitor distance if MOVES CO emissions were assumed to be unbiased. We note here that assuming unbiased CO emissions is not recommended and that applying observationally-derived ΔCO:ΔNO_x_ to constrain NO_x_ emissions must be preceded by an evaluation of CO bias. For instance, past studies of CO in Baltimore have found a 15% overprediction of emissions during summer [[23]] and a factor of 2 overprediction in winter [[33]]. However, since we have no specific information on CO bias here without performing air quality modeling, we assume no CO bias to illustrate the range of NO_x_ scaling factors that can be inferred based on choice of methods and monitor distance alone. We show results both using mean values and using median values. Looking at the means, the results infer a NO_x_ emissions bias in MOVES ranging from no significant bias to a significant 89% high bias. Looking at the medians, the range is from no significant bias to a significant 78% high bias. While the MOVES emissions ratios are not statistically different from the cross-gradient and OLS estimates at 25m, the MOVES model does not capture the highest observed values or the temporal variability in ΔCO:ΔNO_x_ (see Figure 2), indicating that the inputs used to run MOVES may represent typical conditions but did not fully capture hour-specific variability in the types of vehicles, age of vehicles and traffic conditions for this section of I-15.

Figures S5 through S8 split out comparisons from Figure 2 by season. Recent literature suggested that MOVES NO_x_ emissions estimates are unbiased in the winter but overpredicted in summer [[33],[54]]. These plots do not show any discernable seasonal bias pattern in observed ΔCO:ΔNO_x_ or MOVES emitted ratios. However, it is hard to draw any definitive conclusions about the seasonal nature of the comparisons in this study due to small sample size once data are split out by season. As shown in Table S4, over half of the datapoints in our analysis come from springtime with only 5–11 hours for comparison in winter and summer depending on the downwind monitor distance (Figures S5 and S7).

There are several potential explanations for why these various observation-based methods might result in different estimates of ΔCO:ΔNO_x_. First, as described in Section 2.3.2, the OLS regression slope can be biased toward zero if there is non-negligible measurement error in the explanatory variable (i.e., the NO_X_ measurement), while the orthogonal regression slope can be biased high if there is a difference in the relative uncertainty in the CO and NO_x_ measurements. Second, any drift in the measurements of CO and NO_x_ could introduce error into the cross-road gradient calculation. While instruments at all sites underwent zero and span procedures nightly and no major drift issues were identified, the drifts that did occur at the upwind and downwind sites are independent from one another and could add uncertainty to results. Third, there may be variability in the sources impacting the four monitors due to small scale eddies not represented by the measured wind direction and variability in activity patterns.

We additionally explore the utility of constraining NOx emissions based on the CO:NO_x_ ratio by comparing emitted ratios from our MOVES simulation with one that used county-level default input values for vehicle age distributions and fuel characteristics. We find that the county-level default MOVES simulations result in both higher CO and higher NO_x_ emissions and a larger ratio of emitted CO:NO_x_ than the field-study specific information used in this study (Table S4). If the OLS or cross-road gradient ratios were used to constrain the county-level default MOVES NO_x_ emissions only, assuming CO was unbiased, then NO_x_ emissions would have to be increased further in order to match the lower observed ΔCO:ΔNO_x_. Thus, the emitted CO:NO_x_ ratio would be brought closer to the emitted ratio from the field-study specific MOVES run by adjusting the NO_x_ emissions upward that were already higher than the field-study specific MOVES estimates. In this test, CO emissions were more sensitive to MOVES input choices than NO_x_, making it a poor assumption that CO emissions are unbiased. This highlights the need to robustly evaluate CO bias before using ΔCO:ΔNO_x_ to constrain NO_x_ emissions or alternatively exploring whether a different co-emitted pollutant, such as CO_2_, is more robust and less sensitive to input assumptions.

## 4. Discussion

Characterizing ΔCO:ΔNO_x_ values from observational data can be useful for identifying important sources of CO and NO_x_ and constraining emissions estimates from these sources. In this study the methods are intended to isolate impacts of the roadway, so it would be expected that the predominant sources would be gasoline and diesel onroad vehicles. The ΔCO:ΔNO_x_ values from this analysis generally fall between 3 and 7 with some outliers. CO:NO_x_ MOVES emissions ratios from onroad diesel vehicles are around 1 and ratios from onroad gasoline vehicles are between 13 and 14 [[35]]. Therefore, values calculated here suggest a mix of diesel and gasoline vehicles. Somewhat higher values at the 300m site might indicate influence from nonroad gasoline equipment which can have emitted CO:NO_x_ ratios in the range of 19–22 [[35]] and may have been operating either at the nearby golf course or airport. While the study design did not include direct accounting of diesel and gasoline vehicles, we estimated the number of light-duty and heavy-duty vehicles based on collected vehicle length information resulting in fraction of light-duty vehicles between 0.80 and 0.92 at all times during the study period with a mean value of 0.89. Based on these fractions, MOVES estimated that heavy duty vehicles emitted 2.3 times more NO_x_ than light-duty vehicles emitted, which is consistent with the relatively low observed ΔCO:ΔNO_x_ values. Despite this general consistency, we found little temporal correlation between the estimated fraction of light-duty vehicles with observationally-derived ΔCO:ΔNO_x_ values (r ≤ 0.33 for all method/monitor combinations). Figure S9 further depicts the lack of correlation between ΔCO:ΔNO_x_ estimates and the fraction of light-duty vehicles. It is possible that the low correlation reported here and shown in Figure S9 are due to fairly low variability in the light-duty fraction which was near 0.9 for the majority of hours included in this analysis.

**Figure 3.**
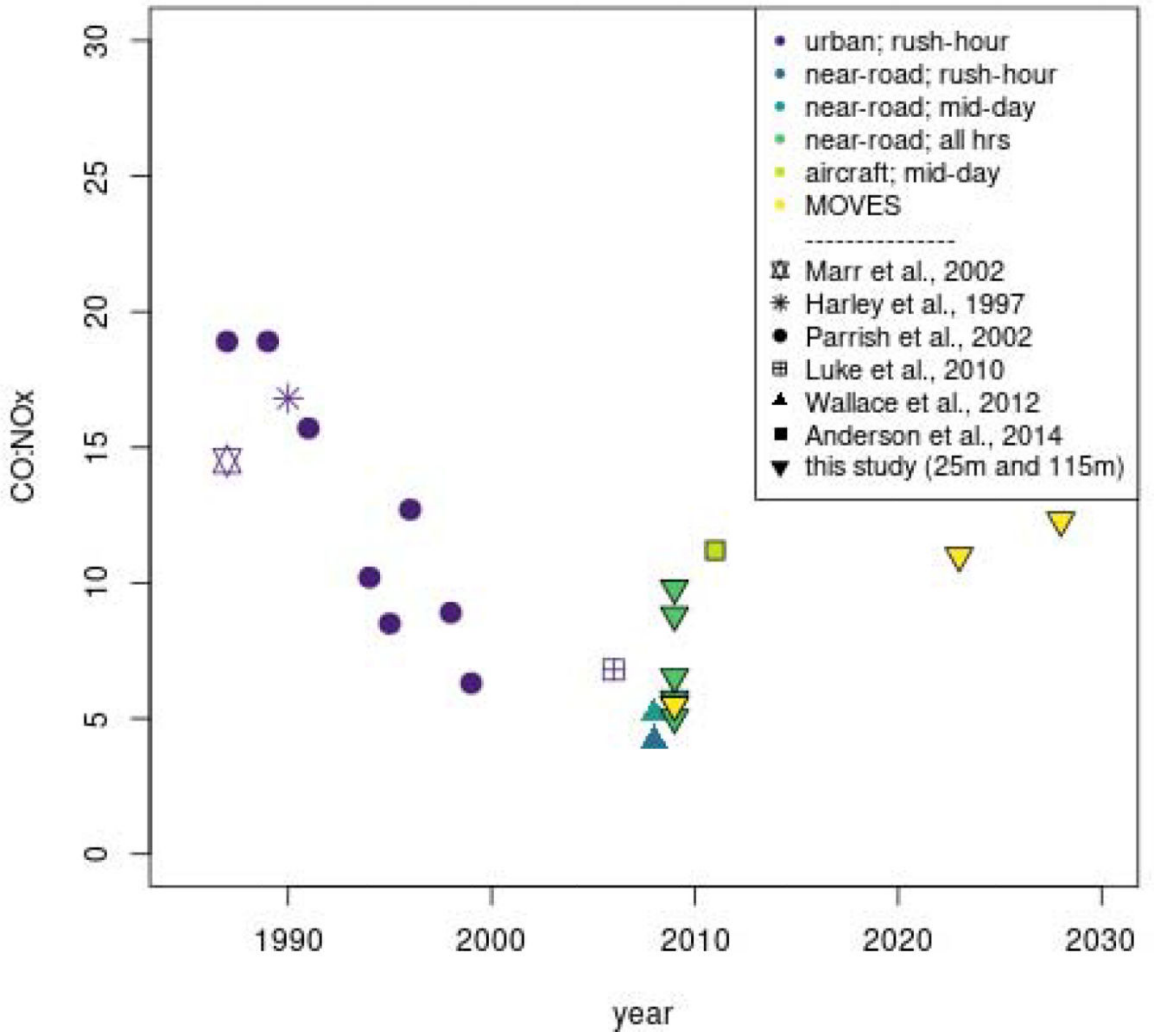
Comparison of regression-based ΔCO:ΔNO_x_ in the literature to median values from each method and results from this study (25m/115m monitor distances and MOVES estimates). The symbol indicates the study from which each datapoint is derived. The colors indicate the type of measurement location and the time-of-day for the measurements.

To further explore whether these methods provide information that is useful for qualitatively identifying emissions sources we compare ratios from this work to those derived in the literature from observational studies (Figure 3; Table S5). ΔCO:ΔNO_x_ values in Figure 3 are color-coded by the method of data screening that they used to isolate a signal from onroad vehicles. Most of the literature values show a decreasing trend over time since the mid 1980s. This decrease over time is likely due to several rounds of vehicle regulations over the past 20 years which have reduced CO and NO_x_ emissions from cars and trucks in the United States at different rates as has also been noted by previous studies [[26,55]]. Specifically, results from Parish [[29]] suggest that the ΔCO:ΔNO_x_ values decreased from 18.9 in 1989 to 8.9 in 1998 in Boulder Colorado and similarly decreased from 10.2 in 1994 to 6.3 in 1999 in Nashville. More recent measurements in Houston [[34]] and Boise [[32]] since 2006 have ΔCO:ΔNO_x_ estimates between 5 and 7 and are consistent with the ΔCO:ΔNO_x_ values inferred from cross-road gradients and OLS in this study but are lower than values inferred from orthogonal regressions reported here. The most recent measurements described by Anderson et al. [[23]] in Baltimore are substantially higher than the ΔCO:ΔNO_x_ values from this study and are similar to values reported in the literature for measurements made in the late 1990s. The higher ΔCO:ΔNO_x_ values from Anderson et al. [[23]] may be caused by chemical aging during the travel of these pollutants due to the fact that measurements were taken in an urban area but not in a near-road environment [[35]]. Comparisons with international studies show much higher ΔCO:ΔNO_x_ values at sites in Mexico City [[24]] and Sao Paulo [[31]] (Table S5). The higher ratios in Mexico City and Sao Paulo may reflect gasoline vehicles that have less stringent emissions controls, similar to what was seen in the earlier US studies. These comparisons suggest that regression-based ΔCO:ΔNO_x_ values can provide qualitative insight into the types of sources affecting near-road locations and the change in those sources over the past several decades. The data in this study, as well as data used in the studies shown in Figure 3 are all at least nine years old and do not represent emissions from the current U.S. vehicle fleet. To better understand how this older data might compare to CO:NO_x_ ratios from onroad vehicles today, we looked at National US emissions estimates available for download from the US EPA[[56]] for 2011, 2014, and 2016, as well as data projected to 2023 and 2028 (we looked at EPA’s “ff” case for all years except 2014 for which the “fd” case was the only case available from the 2011v63_2014v71_2016v1_country-SCC_summary_21-Feb-2020.zip file). These data are derived from MOVES simulations. Taking only onroad running vehicle emissions, we found that the 2011 values of 5.8 is predicted to have increased to 6.2 and 6.9 in 2014 and 2016, respectively, and is expected to increase further by 2023 and 2028 to 11.0 and 12.3. It is important to note that while the CO:NO_x_ ratio is predicted to have increased since 2011, the total emissions of CO and NO_x_ are both predicted to have declined from this source category. This predicted change in CO:NO_x_ for the US fleet could be the results of several different factors. First, it is possible that the relative mix of diesel and gasoline vehicles is predicted to change. A larger fraction of gasoline vehicles on the road would result in higher MOVES estimates of CO:NO_x_. Another possibility is that newer vehicle emissions technologies taking effect in this time period have resulted in larger reductions in NO_x_ than in CO relative to emissions control technologies that were dominant in vehicles operating during 2011.

## 5. Conclusions

This study explores uncertainties in near-road emissions constraints using common methodologies. Using a variety of methods with near road measurements we can infer a wide range of possible top-down constraints on MOVES NO_x_ emissions ranging from unbiased to almost a factor of 2 high-bias. As discussed above, OLS may underestimate slope and orthogonal regression may overestimate slope depending on the relative magnitudes of uncertainties in the dependent and independent variables. Unless one method can be argued to be more appropriate, this suggests the underlying uncertainty in applying these methods may be much larger than suggested by quantitative error estimates (i.e., uncertainty bounds for regression slopes). While we applied two regression methods, OLS and orthogonal, which are commonly applied for estimating CO:NO_x_ ratios, there are other errors in measurement regression methods that could be considered. More flexible methods, such as York regression [[43]], can account for differences in error variance in each individual measurement, as well as correlation between the x and y measurements, if such information is available from the measurement study. In addition, the study used data collected with instruments similar to those currently used routinely in EPA’s near-road monitoring network. However, we expect that the instrument precision and relative error for NO_x_ versus CO may look different for measurements made as part of other special field studies using instrumentation with greater precision, accuracy, and more frequent and/or improved calibrations. Since we have shown that the regression methods are sensitive to the specific measurement uncertainty of a particular dataset, results here may not represent the range of values that would be derived from other specialized field study measurements.

Despite the limitations, near-road constraints represent one of only a few methods to constrain on-road NO_x_ emissions. Additional types of measurement datasets which might not be subject to these same types of uncertainty are available for constraining real-world vehicle emissions estimates. Those measurements include Portable Emissions Measurement Systems (PEMS), which record CO and NO_x_ concentrations from the tailpipe while a vehicle is driving [[57–63]], chaser car studies in which a vehicle tails other vehicles on the road to measure emissions during driving conditions [[64–68]], and remote sensing techniques in which an instrument with an infrared source and corresponding detector situated on opposite sides of a road or freeway on-ramp can intercept exhaust streams from individual vehicles [[30,69–74]].

The use of bottom-up versus top-down inventory methods will depend on the needs of a specific application. Bottom-up inventories provide detailed information on emissions from different source types which may be necessary for regulatory applications. In addition, bottom-up inventories allow for projecting emissions into the future taking into account the impact of new or future regulations which may not be possible with observation-based estimates. Finally, bottom-up inventories may be able to provide a higher spatial and temporal resolution than top-down inventories when suitable inputs are available. However, as shown in the comparison of emissions estimates from MOVES when using county-level default inputs versus field-study specific information on traffic and vehicle fleet, the emissions estimates from MOVES or other bottom-up techniques depend on the accuracy and resolution of input data. Observation-based emissions estimates may be appropriate for some applications especially when quantifying total emissions magnitudes is more important than characterizing the break-out of emissions by source type or activity data. In addition, in cases where accurate input data (emissions factors, activity data, vehicles fleet mix, etc.) are not available, observation-based emissions estimates may be a more appropriate option. Ideally, top-down and bottom-up inventories can work in concert such that bottom-up inventories can provide detailed break-outs of contributions by source type while top-down methods can validate emissions magnitudes and identify cases where assumptions in bottom-up inventories fail.

## Supplementary Material

Sup1

## Figures and Tables

**Table 1.** Summary of underlying error variance assumptions of the methods considered in this analysis. σ_x_ and σ_y_ are the standard deviation of the error in measurements of CO and NO_x_, respectively.

Method	Error in Measurement Assumptions
Cross-road gradient	No instrument drift; differences in up-wind and down-wind monitors are solely due to emissions along I-15
OLS	σ_x_ = 0, or σ_x_ << σ_y_
Orthogonal Regression	σ_x_ / σ_y_ = 1

**Table 2.** MOVES NO_x_ emissions scaling factors based on each ambient data method uncorrected for CO bias. Statistically significant results shown in bold font.

Method	Distance from Roadway (m)	Mean		Median	

		Scaling Ratio	*p*-val*	Scaling Ratio	*p*-val*
**Cross-road gradient**	25	0.94	0.43	0.96	0.92
	115	0.90	0.25	0.99	0.62
	300	0.75	0.10	1.15	0.29

**OLS regression**	25	0.93	0.47	1.09	0.88
	115	**0.80**	**0.02**	0.84	0.06
	300	**0.73**	**4.59 × 10**^**−3**^	**0.90**	**0.04**

**Orthogonal regression**	25	**0.59**	**1.42 × 10**^**−6**^	**0.62**	**1.58 × 10**^**−7**^
	115	**0.56**	**8.23 × 10**^**−7**^	**0.56**	**2.14 × 10**^**−7**^
	300	**0.53**	**2.83 × 10**^**−6**^	**0.67**	**1.36 × 10**^**−8**^

* *P*-values for statistical significance (i.e., statistically different from 1) of the mean-based scaling factors are based on Welsh t-test. P-values for statistical significance the median-based scaling factors are based on Mann Whitney U test. Values in bold indicate statistically significant results (*P* < 0.05).

## References

[1] USEPA. 2014 National Emissions Inventory, version 2 Technical Support Document. OAQPS, Ed. RTP, NC, USA, 2018.

[2] Bond TC; Streets DG; Yarber KF; Nelson SM; Woo JH; Klimont Z A technology-based global inventory of black and organic carbon emissions from combustion. J. Geophys. Res.-Atmos 2004, 109, 43, doi:10.1029/2003jd003697.

[3] NARSTO. Improving Emission Inventories for Effective Air Quality Management across North America. NARSTO: 2005; Vol. NARSTO-05–001.

[4] Simon H; Allen DT; Wittig AE Fine particulate matter emissions inventories: Comparisons of emissions estimates with observations from recent field programs. J. Air Waste Manage. Assoc 2008, 58, 320–343, doi:10.3155/1047-3289.58.2.320. 18318344

[5] Smith SJ; van Aardenne J; Klimont Z; Andres RJ; Volke A; Arias SD Anthropogenic sulfur dioxide emissions: 1850–2005. Atmos. Chem. Phys 2011, 11, 1101–1116, doi:10.5194/acp-11-1101-2011.

[6] Zhao Y; Nielsen CP; Lei Y; McElroy MB; Hao J Quantifying the uncertainties of a bottom-up emission inventory of anthropogenic atmospheric pollutants in China. Atmos. Chem. Phys 2011, 11, 2295–2308, doi:10.5194/acp-11-2295-2011.

[7] MOVES. Availabe online: https://www.epa.gov/moves (accessed on Aug 1, 2019).

[8] EMFAC. Availabe online: https://ww3.arb.ca.gov/msei/msei.htm (accessed on Aug 1, 2019).

[9] Beirle S; Boersma KF; Platt U; Lawrence MG; Wagner T Megacity Emissions and Lifetimes of Nitrogen Oxides Probed from Space. Science 2011, 333, 1737–1739, doi:10.1126/science.1207824. 21940891

[10] Gordon GE Recptor Models. Environ. Sci. Technol 1980, 14, 792–800, doi:10.1021/es60167a006.

[11] Hopke PK; Gladney ES; Gordon GE; Zoller WH; Jones AG Use of Multivariate-Analysis to Identify Sources of Selected Elements in Boston Urban Aerosol. Atmos. Environ 1976, 10, 1015–1025, doi:10.1016/0004-6981(76)90211-0. 1008915

[12] Martin RV; Jacob DJ; Chance K; Kurosu TP; Palmer PI; Evans MJ Global inventory of nitrogen oxide emissions constrained by space-based observations of NO2 columns. J. Geophys. Res.-Atmos 2003, 108, 12, doi:10.1029/2003jd003453.

[13] Schauer JJ; Rogge WF; Hildemann LM; Mazurek MA; Cass GR; Simoneit BRT Source apportionment of airborne particulate matter using organic compounds as tracers. Atmos. Environ 1996, 30, 3837–3855, doi:10.1016/1352-2310(96)00085-4.

[14] Escribano J; Boucher O; Chevallier F; Huneeus N Impact of the choice of the satellite aerosol optical depth product in a sub-regional dust emission inversion. Atmos. Chem. Phys 2017, 17, 7111–7126, doi:10.5194/acp-17-7111-2017.

[15] Kemball-Cook S; Yarwood G; Johnson J; Dornblaser B; Estes M Evaluating NOx emission inventories for regulatory air quality modeling using satellite and air quality model data. Atmos. Environ 2015, 117, 1–8, doi:10.1016/j.atmosenv.2015.07.002.

[16] Lee C; Martin RV; van Donkelaar A; Lee H; Dickerson RR; Hains JC; Krotkov N; Richter A; Vinnikov K; Schwab JJ SO2 emissions and lifetimes: Estimates from inverse modeling using in situ and global, space-based (SCIAMACHY and OMI) observations. J. Geophys. Res.-Atmos 2011, 116, 13, doi:10.1029/2010jd014758.

[17] Buhr M; Parrish D; Elliot J; Holloway J; Carpenter J; Goldan P; Kuster W; Trainer M; Montzka S; McKeen S, Evaluation of ozone precursor source types using principal component analysis of ambient air measurements in rural Alabama. J. Geophys. Res.-Atmos 1995, 100, 22853–22860, doi:10.1029/95jd01837.

[18] Buhr MP; Trainer M; Parrish DD; Sievers RE; Fehsenfeld FC Assessment of pollutant emission inventories by principal component analysis of ambient air measurements. Geophys. Res. Lett 1992, 19, 1009–1012, doi:10.1029/92gl01020.

[19] Fujita EM; Croes BE; Bennett CL; Lawson DR; Lurmann FW; Main HH Comparison of emission inventory and ambient concentration ratios of CO, NMOG, and NOX in California South Coast Air Basin. J. Air Waste Manage. Assoc 1992, 42, 264–276, doi:10.1080/10473289.1992.10466989.

[20] Guo H; Jiang F; Cheng HR; Simpson IJ; Wang XM; Ding AJ; Wang TJ; Saunders SM; Wang T; Lam SHM, Concurrent observations of air pollutants at two sites in the Pearl River Delta and the implication of regional transport. Atmos. Chem. Phys 2009, 9, 7343–7360, doi:10.5194/acp-9-7343-2009.

[21] Li SM; Anlauf KG; Wiebe HA; Bottenheim JW; Shepson PB; Biesenthal T Emission ratios and photochemical production efficiencies of nitrogen oxides, ketones, and aldehydes in the Lower Fraser Valley during the summer Pacific 1993 oxidant study. Atmos. Environ 1997, 31, 2037–2048, doi:10.1016/s1352-2310(96)00126-4.

[22] Mellios G; Van Aalst R; Samaras Z Validation of road traffic urban emission inventories by means of concentration data measured at air quality monitoring stations in Europe. Atmos. Environ 2006, 40, 7362–7377, doi:10.1016/j.atmosenv.2006.06.044.

[23] Anderson DC; Loughner CP; Diskin G; Weinheimer A; Canty TP; Salawitch RJ; Worden HM; Fried A; Mikoviny T; Wisthaler A, Measured and modeled CO and NOy in DISCOVER-AQ: An evaluation of emissions and chemistry over the eastern US. Atmos. Environ 2014, 96, 78–87, doi:10.1016/j.atmosenv.2014.07.004.

[24] Arriaga-Colina JL; West JJ; Sosa G; Escalona SS; Ordunez RM; Cervantes ADM Measurements of VOCs in Mexico City (1992–2001) and evaluation of VOCs and CO in the emissions inventory. Atmos. Environ 2004, 38, 2523–2533, doi:10.1016/j.atmosenv.2004.01.033.

[25] Harley RA; Sawyer RF; Milford JB Updated photochemical modeling for California’s South Coast Air Basin: Comparison of chemical mechanisms and motor vehicle emission inventories. Environ. Sci. Technol 1997, 31, 2829–2839, doi:10.1021/es9700562.

[26] Hassler B; McDonald BC; Frost GJ; Borbon A; Carslaw DC; Civerolo K; Granier C; Monks PS; Monks S; Parrish DD, Analysis of long-term observations of NOx and CO in megacities and application to constraining emissions inventories. Geophys. Res. Lett 2016, 43, 9920–9930, doi:10.1002/2016gl069894.

[27] Kourtidis KA; Ziomas IC; Rappenglueck B; Proyou A; Balis D Evaporative traffic hydrocarbon emissions, traffic CO and speciated HC traffic emissions from the city of Athens. Atmos. Environ 1999, 33, 3831–3842, doi:10.1016/s1352-2310(98)00395-1.

[28] Marr LC; Black DR; Harley RA Formation of photochemical air pollution in central California - 1. Development of a revised motor vehicle emission inventory. J. Geophys. Res.-Atmos 2002, 107, 9, doi:10.1029/2001jd000689.

[29] Parrish DD Critical evaluation of US on-road vehicle emission inventories. Atmos. Environ 2006, 40, 2288–2300, doi:10.1016/j.atmosenv.2005.11.033.

[30] Pierson WR; Gertler AW; Bradow RL Comparison of the SCAQS tunnel study with other on-road vehicle emission data. J. Air Waste Manage. Assoc 1990, 40, 1495–1504, doi:10.1080/10473289.1990.10466799.

[31] Vivanco MG; Andrade MD Validation of the emission inventory in the Sao Paulo Metropolitan Area of Brazil, based on ambient concentrations ratios of CO, NMOG and NOx and on a photochemical model. Atmos. Environ 2006, 40, 1189–1198, doi:10.1016/j.atmosenv.2005.10.041.

[32] Wallace HW; Jobson BT; Erickson MH; McCoskey JK; VanReken TM; Lamb BK; Vaughan JK; Hardy RJ; Cole JL; Strachan SM, Comparison of wintertime CO to NOx ratios to MOVES and MOBILE6.2 on-road emissions inventories. Atmos. Environ 2012, 63, 289–297, doi:10.1016/j.atmosenv.2012.08.062.

[33] Salmon OE; Shepson PB; Ren X; He H; Hall DL; Dickerson RR; Stirm BH; Brown SS; Fibiger DL; McDuffie EE, Top-Down Estimates of NOx and CO Emissions From Washington, DC-Baltimore During the WINTER Campaign. J. Geophys. Res.-Atmos 2018, 123, 7705–7724, doi:10.1029/2018jd028539.

[34] Luke WT; Kelley P; Lefer BL; Flynn J; Rappengluck B; Leuchner M; Dibb JE; Ziemba LD; Anderson CH; Buhr M Measurements of primary trace gases and NOy composition in Houston, Texas. Atmos. Environ 2010, 44, 4068–4080, doi:10.1016/j.atmosenv.2009.08.014.

[35] Simon H; Valin LC; Baker KR; Henderson BH; Crawford JH; Pusede SE; Kelly JT; Foley KM; Chris Owen R; Cohen RC, Characterizing CO and NOy Sources and Relative Ambient Ratios in the Baltimore Area Using Ambient Measurements and Source Attribution Modeling. J. Geophys. Res.-Atmos 2018, 123, 3304–3320, doi:10.1002/2017jd027688. PMC9364951 35958736

[36] Kimbrough S; Baldauf RW; Hagler GSW; Shores RC; Mitchell W; Whitaker DA; Croghan CW; Vallero DA Long-term continuous measurement of near-road air pollution in Las Vegas: seasonal variability in traffic emissions impact on local air quality. Air Qual. Atmos. Health 2013, 6, 295–305, doi:10.1007/s11869-012-0171-x.

[37] Kimbrough S; Hanley T; Hagler G; Baldauf R; Snyder M; Brantley H Influential factors affecting black carbon trends at four sites of differing distance from a major highway in Las Vegas. Air Qual. Atmos. Health 2018, 11, 181–196, doi:10.1007/s11869-017-0519-3. PMC7359888 32665795

[38] Browne EC; Cohen RC Effects of biogenic nitrate chemistry on the NOx lifetime in remote continental regions. Atmos. Chem. Phys 2012, 12, 11917–11932, doi:10.5194/acp-12-11917-2012.

[39] de Foy B; Lu ZF; Streets DG; Lamsal LN; Duncan BN Estimates of power plant NOx emissions and lifetimes from OMI NO2 satellite retrievals. Atmos. Environ 2015, 116, 1–11, doi:10.1016/j.atmosenv.2015.05.056.

[40] Kunhikrishnan T; Lawrence MG; von Kuhlmann R; Richter A; Ladstatter-Weissenmayer A; Burrows JP Analysis of tropospheric NOx over Asia using the model of atmospheric transport and chemistry (MATCH-MPIC) and GOME-satellite observations. Atmos. Environ 2004, 38, 581–596, doi:10.1016/j.atmosenv.2003.09.074.

[41] Liu F; Beirle S; Zhang Q; Dorner S; He KB; Wagner T NOx lifetimes and emissions of cities and power plants in polluted background estimated by satellite observations. Atmos. Chem. Phys 2016, 16, 5283–5298, doi:10.5194/acp-16-5283-2016.

[42] Schaub D; Brunner D; Boersma KF; Keller J; Folini D; Buchmann B; Berresheim H; Staehelin J SCIAMACHY tropospheric NO2 over Switzerland: estimates of NOx lifetimes and impact of the complex Alpine topography on the retrieval. Atmos. Chem. Phys 2007, 7, 5971–5987, doi:10.5194/acp-7-5971-2007.

[43] York D; Evensen NM; Martinez ML; Delgado JD Unified equations for the slope, intercept, and standard errors of the best straight line. Am. J. Phys 2004, 72, 367–375, doi:10.1119/1.1632486.

[44] Prather MJ Time scales in atmospheric chemistry: Theory, GWPs for CH4 and CO, and runaway growth. Geophys. Res. Lett 1996, 23, 2597–2600, doi:10.1029/96gl02371.

[45] Xiao YP; Jacob DJ; Turquety S Atmospheric acetylene and its relationship with CO as an indicator of air mass age. J. Geophys. Res.-Atmos 2007, 112, 14, doi:10.1029/2006jd008268.

[46] Barre J; Edwards D; Worden H; Arellano A; Gaubert B; Da Silva A; Lahoz W; Anderson J On the feasibility of monitoring carbon monoxide in the lower troposphere from a constellation of northern hemisphere geostationary satellites: Global scale assimilation experiments (Part II). Atmos. Environ 2016, 140, 188–201, doi:10.1016/j.atmosenv.2016.06.001. PMC6999668 32021559

[47] Edwards DP; Emmons LK; Hauglustaine DA; Chu DA; Gille JC; Kaufman YJ; Petron G; Yurganov LN; Giglio L; Deeter MN, Observations of carbon monoxide and aerosols from the Terra satellite: Northern Hemisphere variability. J. Geophys. Res.-Atmos 2004, 109, 17, doi:10.1029/2004jd004727.

[48] Wu C; Yu JZ Evaluation of linear regression techniques for atmospheric applications: the importance of appropriate weighting. Atmos. Meas. Tech 2018, 11, 1233–1250, doi:10.5194/amt-11-1233-2018.

[49] Cantrell CA Technical Note: Review of methods for linear least-squares fitting of data and application to atmospheric chemistry problems. Atmos. Chem. Phys 2008, 8, 5477–5487, doi:10.5194/acp-8-5477-2008.

[50] R_Core_Team R: A language and environment for statistical computing., R Foundation for Statistical Computing: Vienna, Austria, 2013.

[51] USEPA. MOVES2014, MOVES2014a, and MOVES2014b Technical Guidance: Using MOVES to Prepare Emission Inventories for State Implementation Plans and Transportation Conformity; Ann Arbor, MI, USA, 2018.

[52] FHA. UC Riverside, Improving Vehicle Fleet, Activity, and Emissions Data for On-Road Mobile Sources Emissions Inventories FINAL REPORT, Prepared for: Federal Highway Administration. FHA: Washigton DC, USA, 2011.

[53] Snyder M Filling the gaps: Estimating roadway emissions using inconsistent traffic measurements in Las Vegas, Nevada near-road field study. In Proceedings of Community Modeling and Analysis System Annual Conference, Chapel Hill, NC, USA, 22–24 October 2018.

[54] Hall DL; Anderson DC; Martin CR; Ren XR; Salawitch RJ; He H; Canty TP; Hains JC; Dickerson RR Using near-road observations of CO, NOy, and CO2 to investigate emissions from vehicles: Evidence for an impact of ambient temperature and specific humidity. Atmos. Environ 2020, 232, 12, doi:10.1016/j.atmosenv.2020.117558.

[55] Parrish DD; Allen DT; Bates TS; Estes M; Fehsenfeld FC; Feingold G; Ferrare R; Hardesty RM; Meagher JF; Nielsen-Gammon JW, Overview of the Second Texas Air Quality Study (TexAQS II) and the Gulf of Mexico Atmospheric Composition and Climate Study (GoMACCS). Journal of Geophysical Research: Atmospheres 2009, 114, D00F13, doi:10.1029/2009jd011842.

[56] USEPA. Emissions Files for 2016 Emissions Modeling Platofrm. Availabe online: ftp://newftp.epa.gov/air/emismod/2016/v1/reports/ (accessed on Sep 1, 2020).

[57] Bishop JDK; Molden N; Boies AM Using portable emissions measurement systems (PEMS) to derive more accurate estimates of fuel use and nitrogen oxides emissions from modern Euro 6 passenger cars under real-world driving conditions. Applied Energy 2019, 242, 942–973, doi:10.1016/j.apenergy.2019.03.047.

[58] Carslaw DC; Beevers SD; Tate JE; Westmoreland EJ; Williams ML Recent evidence concerning higher NOx emissions from passenger cars and light duty vehicles. Atmos. Environ 2011, 45, 7053–7063, doi:10.1016/j.atmosenv.2011.09.063.

[59] Durbin TD; Johnson K; Cocker DR; Miller JW; Maldonado H; Shah A; Ensfield C; Weaver C; Akard M; Harvey N, Evaluation and Comparison of Portable Emissions Measurement Systems and Federal Reference Methods for Emissions from a Back-Up Generator and a Diesel Truck Operated on a Chassis Dynamometer. Environ. Sci. Technol 2007, 41, 6199–6204, doi:10.1021/es0622251. 17937302

[60] Durbin TD; Johnson K; Miller JW; Maldonado H; Chernich D Emissions from heavy-duty vehicles under actual on-road driving conditions. Atmos. Environ 2008, 42, 4812–4821, doi:10.1016/j.atmosenv.2008.02.006.

[61] Giechaskiel B; Clairotte M; Valverde-Morales V; Bonnel P; Kregar Z; Franco V; Dilara P Framework for the assessment of PEMS (Portable Emissions Measurement Systems) uncertainty. Environmental Research 2018, 166, 251–260, doi:10.1016/j.envres.2018.06.012. 29908456 PMC6143386

[62] Johnson KC; Durbin TD; Cocker DR; Miller WJ; Bishnu DK; Maldonado H; Moynahan N; Ensfield C; Laroo CA On-road comparison of a portable emission measurement system with a mobile reference laboratory for a heavy-duty diesel vehicle. Atmos. Environ 2009, 43, 2877–2883, doi:10.1016/j.atmosenv.2009.03.019.

[63] Mamakos A; Bonnel P; Perujo A; Carriero M Assessment of portable emission measurement systems (PEMS) for heavy-duty diesel engines with respect to particulate matter. J. Aerosol. Sci. 2013, 57, 54–70, doi:10.1016/j.jaerosci.2012.10.004.

[64] Kittelson DB; Watts WF; Johnson JP; Schauer JJ; Lawson DR On-road and laboratory evaluation of combustion aerosols—Part 2:: Summary of spark ignition engine results. J. Aerosol. Sci 2006, 37, 931–949, doi:10.1016/j.jaerosci.2005.08.008.

[65] An F; Barth M; Ross M Vehicle Total Life-Cycle Exhaust Emissions. In Proceedings of Total Life Cycle Conference and Exposition, Vienna, Austria, 1 October, 1995.

[66] Morawska L; Ristovski; Johnson; Jayaratne; Mengersen K. Novel Method for On-Road Emission Factor Measurements Using a Plume Capture Trailer. Environ. Sci. Technol. 2007, 41, 574–579, doi:10.1021/es060179z. 17310724

[67] Wehner B; Uhrner U; von Löwis S; Zallinger M; Wiedensohler A Aerosol number size distributions within the exhaust plume of a diesel and a gasoline passenger car under on-road conditions and determination of emission factors. Atmos. Environ 2009, 43, 1235–1245, doi:10.1016/j.atmosenv.2008.11.023.

[68] Canagaratna MR; Jayne JT; Ghertner DA; Herndon S; Shi Q; Jimenez JL; Silva PJ; Williams P; Lanni T; Drewnick F, Chase Studies of Particulate Emissions from in-use New York City Vehicles. Aerosol Science and Technology 2004, 38, 555–573, doi:10.1080/02786820490465504.

[69] Burgard DA; Bishop GA; Stedman DH; Gessner VH; Daeschlein C Remote Sensing of In-Use Heavy-Duty Diesel Trucks. Environ. Sci. Technol 2006, 40, 6938–6942, doi:10.1021/es060989a. 17153998

[70] Bishop GA; Starkey JR; Ihlenfeldt A; Williams WJ; Stedman DH IR Long-Path Photometry: A Remote Sensing Tool for Automobile Emissions. Analytical Chemistry 1989, 61, 671A–677A, doi:10.1021/ac00185a746. 2473669

[71] Zhang Y; Stedman DH; Bishop GA; Guenther PL; Beaton SP Worldwide On-Road Vehicle Exhaust Emissions Study by Remote Sensing. Environ. Sci. Technol 1995, 29, 2286–2294, doi:10.1021/es00009a020. 22280268

[72] Popp PJ; Bishop GA; Stedman DH Development of a High-Speed Ultraviolet Spectrometer for Remote Sensing of Mobile Source Nitric Oxide Emissions. J. Air Waste Manage. Assoc 1999, 49, 1463–1468, doi:10.1080/10473289.1999.10463978. 28060639

[73] Guenther PL; Bishop GA; Peterson JE; Stedman DH Emissions from 200 000 vehicles: a remote sensing study. Science of The Total Environment 1994, 146–147, 297–302, doi:10.1016/0048-9697(94)90249-6.

[74] Burgard DA; Bishop GA; Stadtmuller RS; Dalton TR; Stedman DH Spectroscopy Applied to On-Road Mobile Source Emissions. Applied Spectroscopy 2006, 60, 135A–148A, doi:10.1366/000370206777412185. 16756695

