# Supplementary material for: Variability in Observation-based Onroad Emission Constraints from a Near-road Environment
Source: Atmosphere (Basel). Author manuscript; Available in PMC 2021 Nov 18. (PMC7821344; doi:10.3390/atmos11111243)
Supplement: Sup1 [file NIHMS1656167-supplement-Sup1.docx]

Supplemental Information for:

Variability in observation-based onroad emission constraints from a near-road environment

Heather Simon^1^, Barron Henderson^1^, R. Chris Owen^1^, Kristen Foley^2^, Michelle G. Snyder^3^, Sue Kimbrough^2^,

^1^Office of Air Quality Planning and Standards, US EPA, RTP, NC

^2^Center for Environmental Measurement and Modeling, US EPA, RTP, NC

^3^Wood Environment and Infrastructure Solutions, Inc., Durham, NC


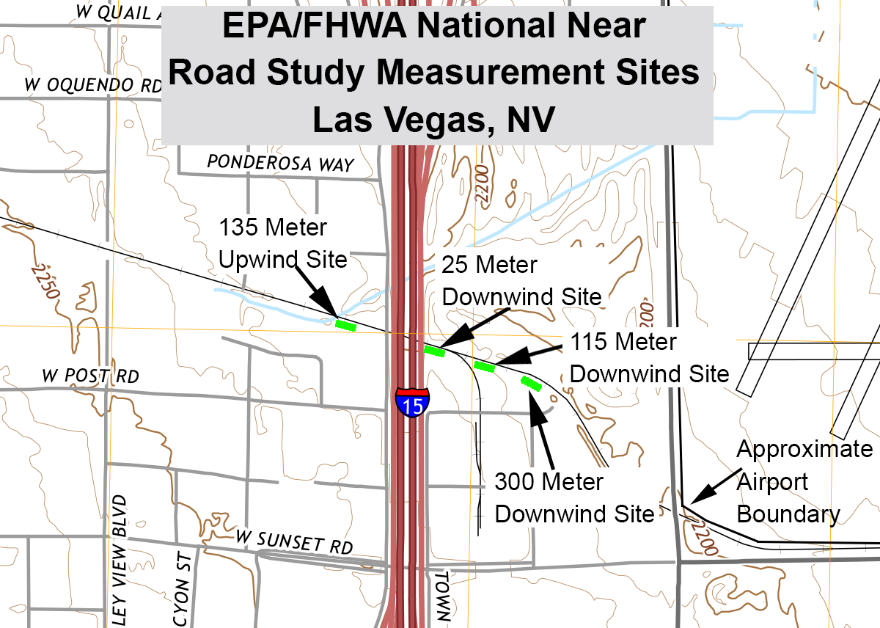


Figure S1. Map of Las Vegas Study Sites


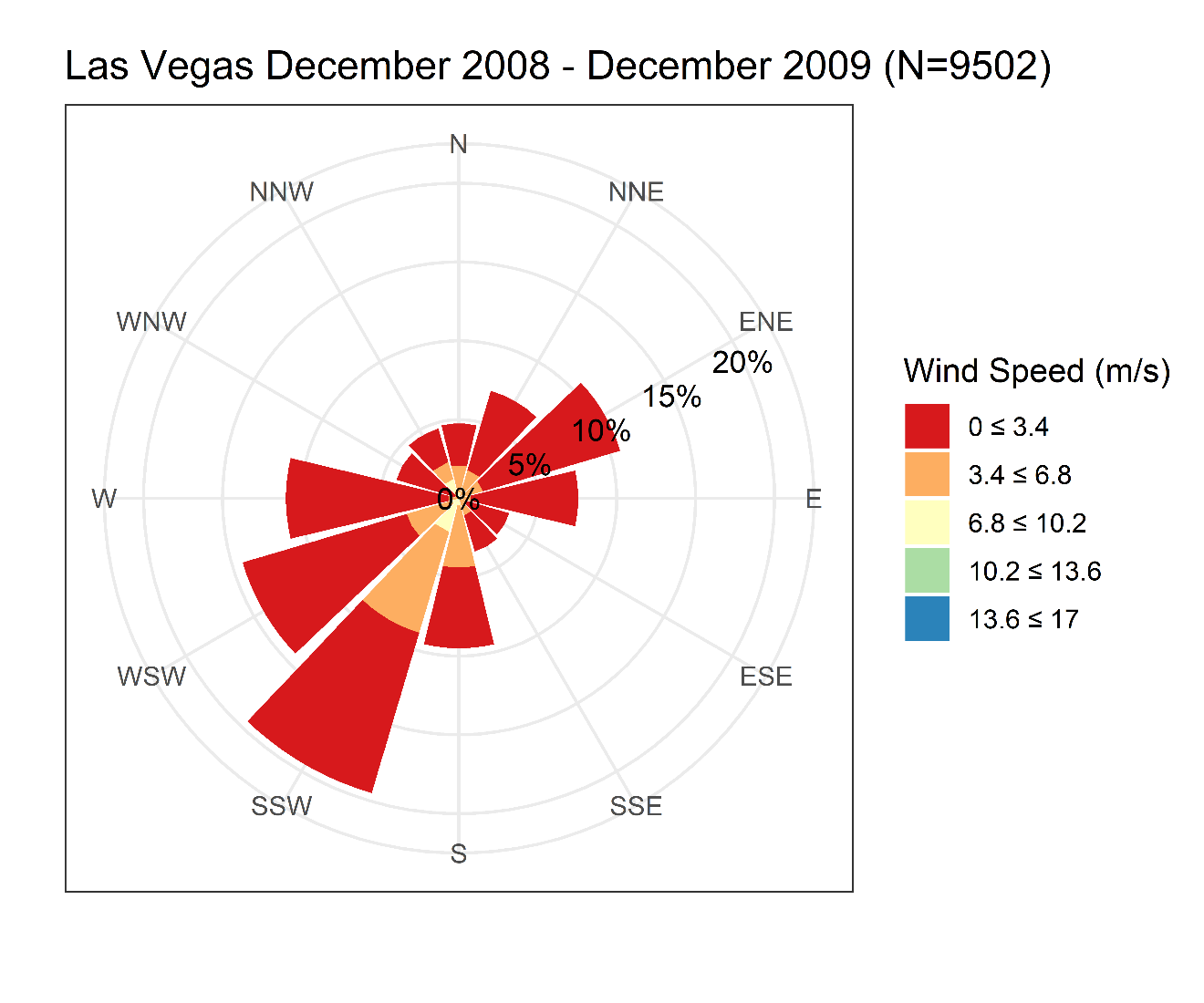


Figure S2. Wind rose for Las Vegas study site from Dec 2008-December 2009.


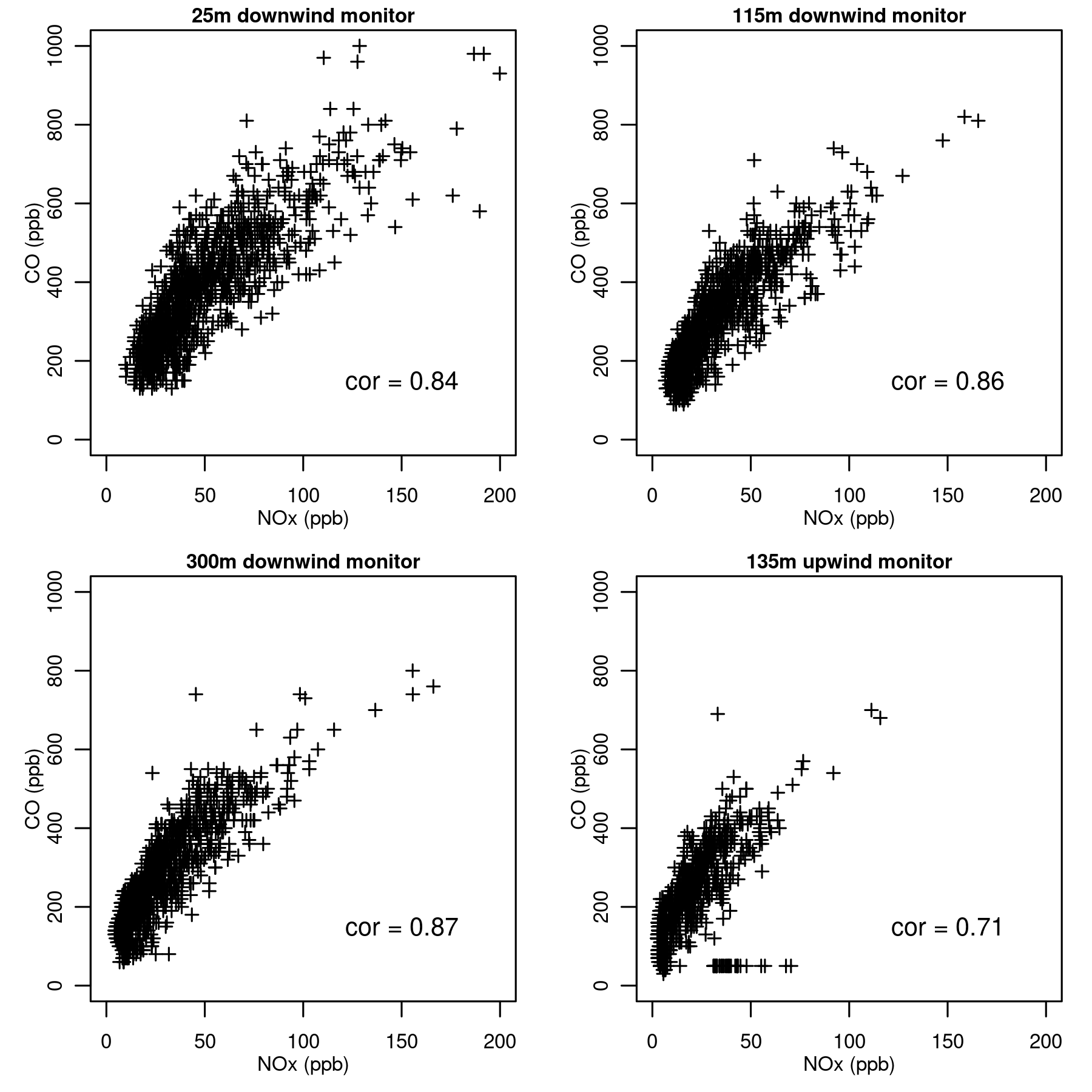


Figure S3. Comparison of 5-minute CO and NO_x_ data for all 168 hours included in this analysis at each monitor location.


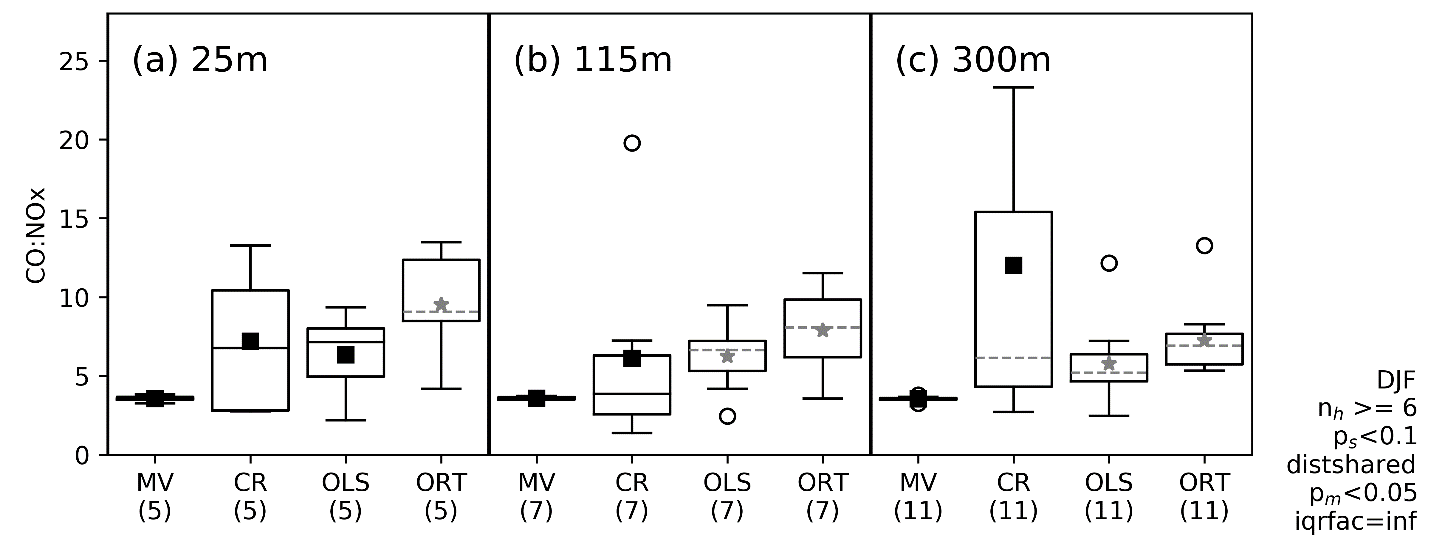


Figure S4. Winter (December, January, February) comparison of ∆CO:∆NOx values from MOVES and 3 ambient-based methods. Distribution of emitted CO:NOx from MOVES (“MV”), (a); Distribution of ∆CO:∆NOx values from the cross-road gradient method (“CR”), at different distances from the roadway (b). Distribution of ∆CO:∆NOx values from OLS regressions and at different distances from the roadway (c). Distribution of ∆CO:∆NOx values from orthogonal regressions (“ORT”) at the 25m downwind monitor (a), the 115m downwind monitor (b) and the 300m downwind monitor (c). different distances from the roadway (d). Numbers below each boxplot represent (n, max value). Sample size excludes outlier values and insignificant regression slopes. Boxes represent interquartile range; mid-lines represent median values; and symbols represent mean values. When the Mann Whitney test is statistically different from MOVES, the median line is grey and dashed. When the Welch’s t-test is statistically different from MOVES, the mean is a star and grey.


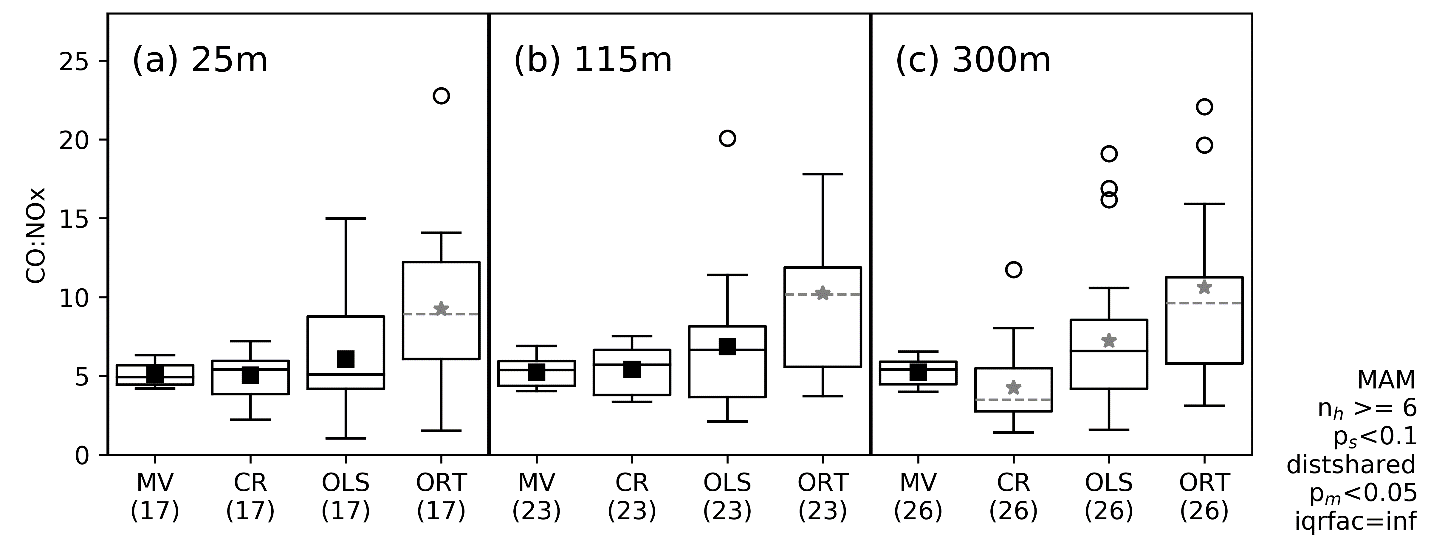


Figure S5. Spring (March, April May) comparison of ∆CO:∆NOx values from MOVES and 3 ambient-based methods. Distribution of emitted CO:NOx from MOVES (“MV”), (a); Distribution of ∆CO:∆NOx values from the cross-road gradient method (“CR”), at different distances from the roadway (b). Distribution of ∆CO:∆NOx values from OLS regressions and at different distances from the roadway (c). Distribution of ∆CO:∆NOx values from orthogonal regressions (“ORT”) at the 25m downwind monitor (a), the 115m downwind monitor (b) and the 300m downwind monitor (c). different distances from the roadway (d). Numbers below each boxplot represent (n, max value). Sample size excludes outlier values and insignificant regression slopes. Boxes represent interquartile range; mid-lines represent median values; and symbols represent mean values. When the Mann Whitney test is statistically different from MOVES, the median line is grey and dashed. When the Welch’s t-test is statistically different from MOVES, the mean is a star and grey.


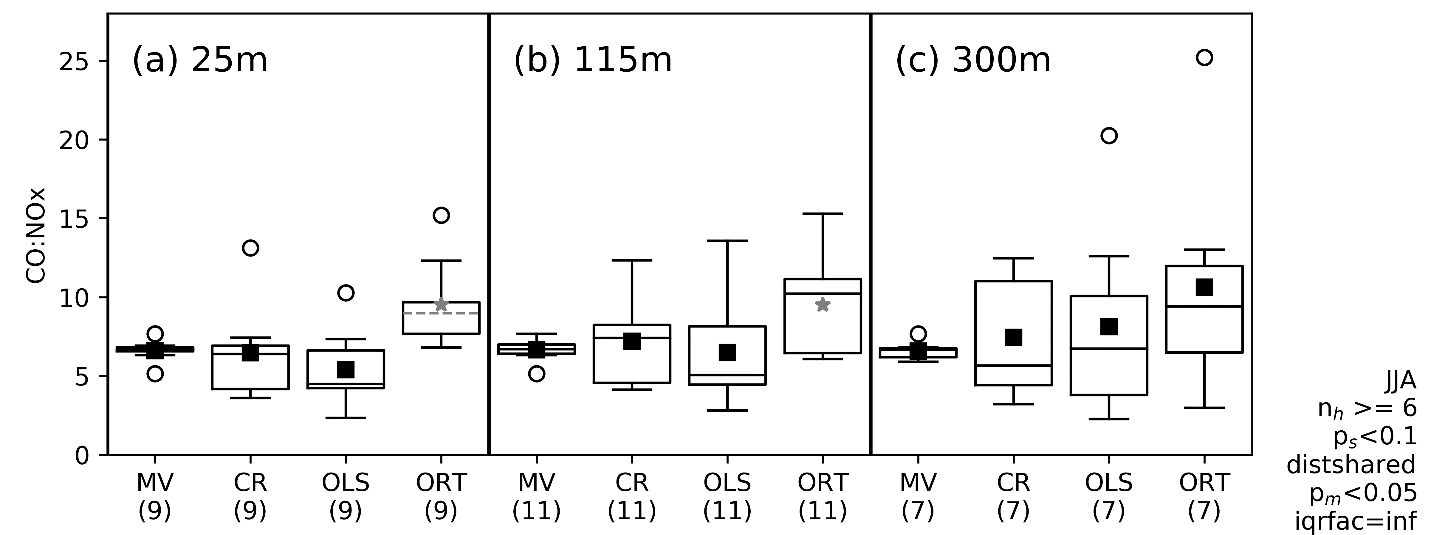


Figure S6. Summer (June, July, August) comparison of ∆CO:∆NOx values from MOVES and 3 ambient-based methods. Distribution of emitted CO:NOx from MOVES (“MV”), (a); Distribution of ∆CO:∆NOx values from the cross-road gradient method (“CR”), at different distances from the roadway (b). Distribution of ∆CO:∆NOx values from OLS regressions and at different distances from the roadway (c). Distribution of ∆CO:∆NOx values from orthogonal regressions (“ORT”) at the 25m downwind monitor (a), the 115m downwind monitor (b) and the 300m downwind monitor (c). different distances from the roadway (d). Numbers below each boxplot represent (n, max value). Sample size excludes outlier values and insignificant regression slopes. Boxes represent interquartile range; mid-lines represent median values; and symbols represent mean values. When the Mann Whitney test is statistically different from MOVES, the median line is grey and dashed. When the Welch’s t-test is statistically different from MOVES, the mean is a star and grey.


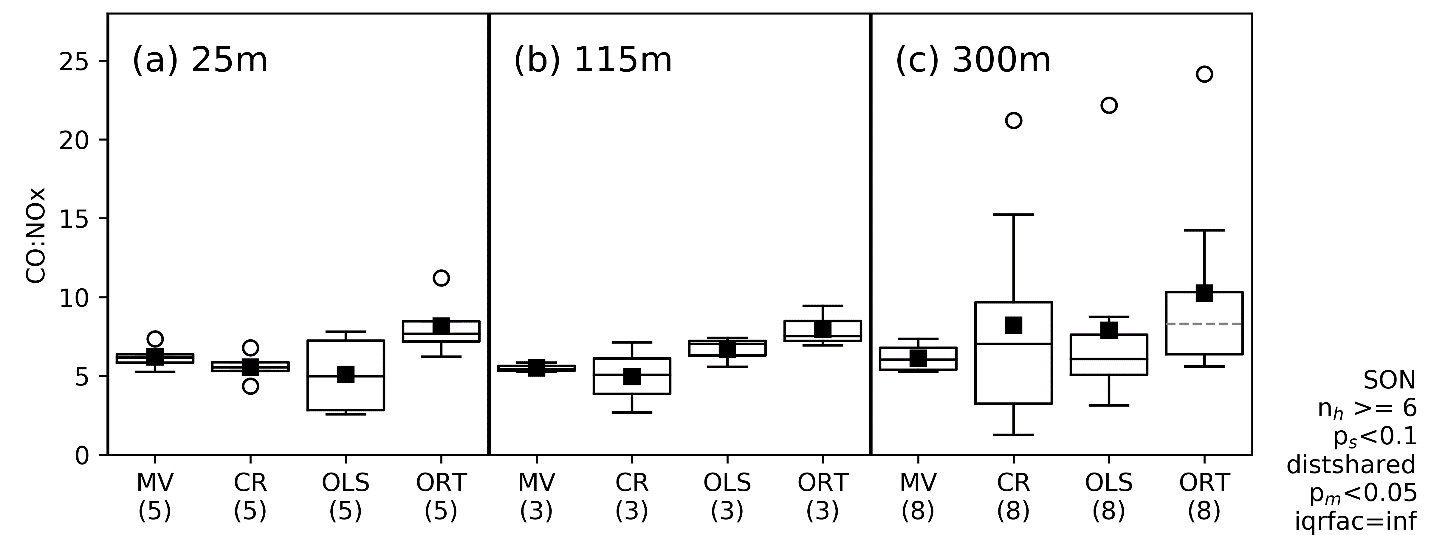


Figure S7. Autumn (September, October, November) comparison of ∆CO:∆NOx values from MOVES and 3 ambient-based methods. Distribution of emitted CO:NOx from MOVES (“MV”), (a); Distribution of ∆CO:∆NOx values from the cross-road gradient method (“CR”), at different distances from the roadway (b). Distribution of ∆CO:∆NOx values from OLS regressions and at different distances from the roadway (c). Distribution of ∆CO:∆NOx values from orthogonal regressions (“ORT”) at the 25m downwind monitor (a), the 115m downwind monitor (b) and the 300m downwind monitor (c). different distances from the roadway (d). Numbers below each boxplot represent (n, max value). Sample size excludes outlier values and insignificant regression slopes. Boxes represent interquartile range; mid-lines represent median values; and symbols represent mean values. When the Mann Whitney test is statistically different from MOVES, the median line is grey and dashed. When the Welch’s t-test is statistically different from MOVES, the mean is a star and grey.


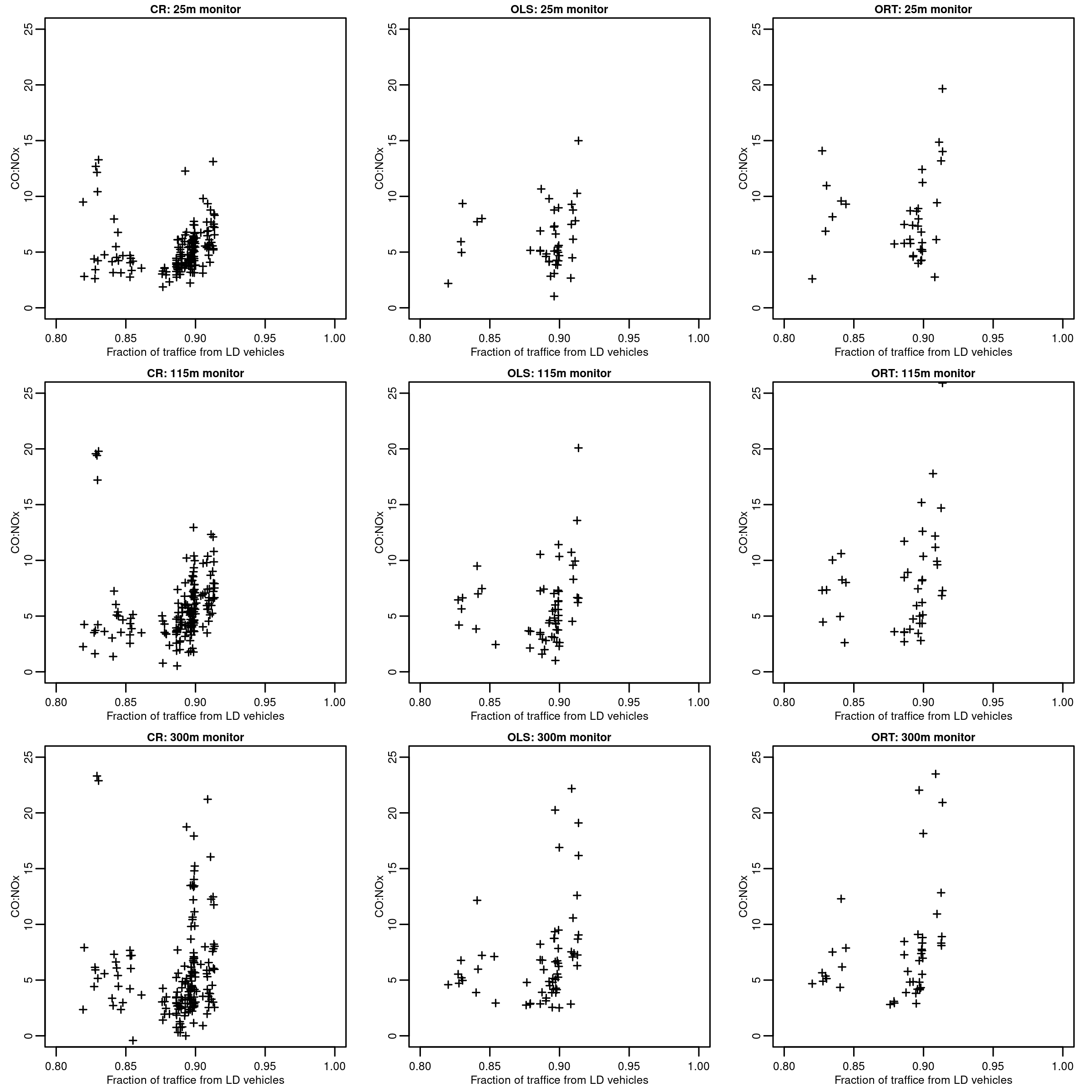


Figure S8.Comparison of ∆CO:∆NOx from all nine ambient datasets (3 methods and 3 downwind monitors) with the fraction of light-duty vehicles estimated on I-15 for the hour of each estimate.

Table S1. Instrument summary and manufacturer specifications

| **Measurement Parameter** | **Sampling Approach** | **Monitoring sites outfitted with instrument** | **Instrument Data** | | | |
| --- | --- | --- | --- | --- | --- | --- |
|  |  |  | **Make/Model** | **Accuracy** | **Precision** | **Detection Limit** |
| **Gas Analyzers** | | | | | | |
| Carbon Monoxide | continuous monitoring  (NDIR FRM CO analyzer) | 100m upwind;  20m downwind;  100m downwind;  300m downwind | EC 9830T | ± 5% 0-1000ppb | 0.5% of reading | 25 ppb |
| Oxides of nitrogen | Chemilluminescence  (FRM analyzer) | 100m upwind;  20m downwind;  100m downwind;  300m downwind | EC 9841B | < 1% | 0.5 ppb | 0.5 ppb |
| **Meteorological Instruments** | | | | | | |
| Wind Speed | Sonic anemometer | 100m upwind;  20m downwind;  100m downwind;  300m downwind | Young Model 81000 | ±0.05 m/s | std. dev. 0.05 m/s at 12 m/s | 0.01 m/s |
| Wind Speed |  |  |  | ± 5° | ± 10° | 0.1° |
| Air Temperature | Temperature probe | 100m downwind | Vaisala HMP45D  Vaisala HMP45A | ±0.2°C at 20° C | 0.1 ° C | 0.1 ° C |
| % Relative Humidity | Relative humidity sensor | 100m downwind |  | ±2%RH from 0…90% RH) | 1% RH | 1% RH |
| Solar Radiation | solar radiation | 100m downwind | MetOne 394 Pyranometer | ±5% from 0…2800 watts meter^2^ | ±1% constancy from -20°C to +40°C | 9 mV/kwatt meter-2, approx |
| **Traffic Data** | | | | | | |
| Vehicle Counts  Vehicle Speed  Vehicle Length Bin | side-fire radar |  | Wavetronix SmartSensor HD |  |  |  |

Table S2. Sample size (number of hours) grouped by time of day and season

| Time of Day | | | |
| --- | --- | --- | --- |
| Morning  (6am-9am) | Day  (10am-4pm) | Evening  (5pm-7pm) | Night  (8pm-5am) |
| 4 | 16 | 51 | 74 |
| Season | | | |
| Spring (Mar/Apr/May) | Summer (Jun/Jul/Aug) | Fall  (Sep/Oct/Nov) | Winter  (Dec/Jan/Feb) |
| 74 | 32 | 14 | 25 |

Table S3. Percentage of regressions with statistically significant slopes

|  | DW Monitor Distance (m) | % of regression fits with a significant slope |
| --- | --- | --- |
|  |  |  |
| Ordinary Least Squares | 20 | 40% |
|  | 100 | 46% |
|  | 300 | 55% |
| Orthogonal | 20 | 29% |
|  | 100 | 32% |
|  | 300 | 38% |

Table S4. Mean and median ∆CO:∆NOx derived in this study

| Method | Distance from roadway (m) | Sample Size | Mean ∆CO:∆NOx | Median ∆CO:∆NOx | Mean emitted CO (mol/m^2^-s) | Mean emitted NOx (mol/m^2^-s) |
| --- | --- | --- | --- | --- | --- | --- |
| MOVES (field site specific inputs) | 25 | 36 | 5.4 | 5.5 | 1339.7 | 248.7 |
|  | 115 | 44 | 5.4 | 5.5 | 1275.7 | 236.9 |
|  | 300 | 52 | 5.2 | 5.4 | 1216.0 | 235.2 |
| MOVES (county default inputs) | 25 | 36 | 6.5 | 6.5 | 2169.0 | 333.2 |
|  | 115 | 44 | 6.4 | 6.5 | 2070.8 | 318.3 |
|  | 300 | 52 | 6.3 | 6.4 | 1968.6 | 313.4 |
| Cross-road | 25 | 36 | 5.8 | 5.7 | N/A | N/A |
|  | 115 | 44 | 5.9 | 5.5 | N/A | N/A |
|  | 300 | 52 | 6.9 | 4.7 | N/A | N/A |
| OLS regression | 25 | 36 | 5.8 | 5.0 | N/A | N/A |
|  | 115 | 44 | 6.7 | 6.5 | N/A | N/A |
|  | 300 | 52 | 7.1 | 6.0 | N/A | N/A |
| Orthogonal regression | 25 | 36 | 9.2 | 8.8 | N/A | N/A |
|  | 115 | 44 | 9.5 | 9.8 | N/A | N/A |
|  | 300 | 52 | 9.8 | 8.1 | N/A | N/A |

Table S5. Regression-based ∆CO:∆NOx from studies in the literature

| **Study** | **Regression Method** | **Location** | **Year** | **∆CO:∆NOx** |
| --- | --- | --- | --- | --- |
| Studies conducted in the United States | | | | |
| Harley et al. (1997) [^23^](#_ENREF_23) | Unknown – CO as explanatory variable | Southern California | 1987 | 16.8* |
| Marr et al. (2002) [^25^](#_ENREF_25) | OLS - CO as explanatory variable | San Francisco | 1990 | 14.5* |
| Parish et al. (2006) [^26^](#_ENREF_26) | Orthogonal Regression using known uncertainty for each instrument | Nashville | 1994 | 10.2 ±1.5 |
|  |  |  | 1995 | 8.5±1.3 |
|  |  |  | 1999 | 6.3±0.9 |
|  |  | Boulder | 1989 | 18.9±2.7 |
|  |  |  | 1991 | 15.7±2.3 |
|  |  |  | 1996 | 12.7±2.0 |
|  |  |  | 1998 | 8.9±1.3 |
|  |  | Los Angeles | 1987 | 18.9±2.1 |
| Luke et al. (2010) [^30^](#_ENREF_30) | Ordinary Least Squares | Houston | 2006 | 6.81±0.94 |
| Wallace et al. (2012) [^29^](#_ENREF_29) | Unknown | Boise | 2008-2009 | 5.2±0.5 |
| Anderson et al. (2014) [^21^](#_ENREF_21) | Orthogonal | Baltimore | 2011 | 11.2±1.2 |
| Studies conducted outside the United States | | | | |
| Kourtidis et al. (1999) [^24^](#_ENREF_24) | Unknown – CO as explanatory variable | Athens | 1994 | 25.2 |
| Ariaga-Colina et al. (2004) [^22^](#_ENREF_22) | Unknown | Mexico City | 1996-2000 | 34.9-42.9 |
| Vivanco et al. (2006) [^28^](#_ENREF_28) | Unknown – CO as explanatory variable | Sao Paulo | 1999 | 14.6* |

*These studies used CO as the explanatory variable and consequently reported ∆NO_x_:∆CO ratios. Values in Table S4 were converted to ∆CO:∆NOx.
